# A Reply to Dr. Kirk’s Article on Coagulation

**Published:** 1894-08

**Authors:** Max Greenbaum

**Affiliations:** Philadelphia


### A REPLY TO DR. KIRK'S ARTICLE ON COAGULATION.

BY MAX GREENBAUM, D.D.S., PHILADELPHIA.

In order that we may arrive at a just conclusion in a controversy, each side presents its testimony and permits counter-criticisms being passed upon it. If one side present a faulty argument, it is proper that the other side should point it out. If there be any misconceptions, they should be set aright. In this way generally we glean the truth and arrive at the common point of harmony in practice. Dr. Kirk, in his article "Coagulants in the Treatment of the Pulp-Chamber and Canals," criticises the views of those who maintain that coagulating applications are detrimental in pulpless teeth as being "mere assumptions" and "serious errors in reasoning," and as a warrant for such criticisms presents a series of experiments. Now, that is a very fair way of proceeding. But if the warrant lack validity, and, furthermore, if the views antagonized have been stated but partially conceived, why, then it is natural that those who have been criticised should desire the presentation of their testimony.

That Dr. Kirk should have assumed that all who refrain from the use of coagulants in the treatment of the root-canal do so because the formed coagulum presents a barrier to any further diffusion of the coagulating application, or any other subsequent application, and that Dr. Harlan was the originator or, at least, the chief exponent of such views, is, to say the least, but a very partial statement, and one that reflects quite seriously upon his understanding of the question.

With some, and they seem to be a large number, the question is not simply whether the coagulating application is a barrier to its own diffusion, but as Dr. Kirk has acknowledged in many instances where canal work is in operation, "nothing can be taken out and nothing can be put in;" and if he feels that his experiments have done anything in the way of disproving "those assumptions" and "serious errors in reasoning," of which he thinks Dr. Harlan is the originator or, at least, chief exponent, we cannot see it in that way.

Dr. Kirk is not thoroughly acquainted with the teachings upon this subject, otherwise Dr. Harlan never would have been placed in the unenviable position of originator or chief exponent. The use of such irritating coagulants as Dr. Kirk mentions in his experiments have been discarded by many in the treatment of the canal for the last twenty-five years, and twenty-five years carry us back farther than 1887.

But, aside from this, he commits himself more seriously, which further sustains our objections.

Dr. Kirk remarks, "The statement of different writers and speakers upon dental therapeutics, to the effect that medicaments which possess the property of forming a coagulum with albuminous organic matter should be excluded from the treatment of pulpless teeth." Now, if he thinks such statement represents the views of those whom he is antagonizing, he may learn otherwise. It is by no means a question of excluding such medicaments as possess the property of forming a coagulum with albuminous matter, and the essayist commits himself in the making of such a statement. The question is, of excluding *irritating coagulants.* Many who refrain from the use of carbolic acid in canal work find in campho-phénique probably one of the best medicaments in the treatment of pulpless teeth, and yet campho-phénique will coagulate albumen.

Then, again, Dr. Kirk remarks, "That the able researches of Professor Miller and Professor Black have thrown so much light upon the antiseptic value of a number of substances which are devoid of coagulating property, and which it has been proposed to utilize instead of coagulants in pulp-canal treatment." Now, if Dr. Kirk believes that such medicaments have been recommended too freely and frequently,—although, very wisely, he fails to mention just what medicaments these are,—it may be well to state that many whom he is criticising recommend coagulating applications in canal work, but that they recognize the *difference* in the coagulation produced between the medicaments which they approve and disapprove, which Dr. Kirk apparently does not seem to know. These facts, taken together, strengthen the observation, to say the least, that he has but partially conceived the views which he regards as "mere assumptions" and "serious errors in reasoning." This rightly questions the strength of his entire arguments. But we may go further, and observe that, even though his conceptions had been complete of the views he is antagonizing, his experiments give him no warrant for any antagonism. In other words, they have nothing to do with what is antagonized; or if he should insist they had, then he commits himself to other serious difficulties, which we shall observe later.

The essayist undertook a series of experiments obviously for the purpose of demonstrating that coagulating applications do not act detrimentally in canals, aside from the fact that many whom he is criticising never assumed such position, as was observed in the use of campho-phénique. Let us say he confines his criticism to those who object to *irritating* medicaments, as carbolic acid or zinc chloride. If his experiments prove, then, that such medicaments do not act detrimentally, they also disprove that which is held by a number of practitioners, that irritating coagulants are detrimental in canal work.

In order to understand the side of the question intended to be disproved, we must have a complete interpretation of it, and that is, when irritating coagulants are employed in canal work, they effect the tissue surrounding the canal, in many cases, but partially, nor have we the warrant for saying that any application acts thoroughly in any case; and this is owing to the minuteness and irregular shapes of a great number of canals; and no application can be worked so well, nor do the conditions under which we work admit of the necessary exactitude that we could say of it, *it has done the work;* and therefore, as a great number of cases admit of no treatment, and others of but partial treatment, and as we have no warrant for saying that any case is thoroughly treated, so have we the right to anticipate future irritation, and this irritation is made more formidable by the use of irritating coagulants.

As Dr. Kirk attempts to disprove that question, what has he done to show this? Nothing. If certain views are to be controverted through means of experiments, those experiments should embrace all the conditions included in that which is to be controverted. His experiments upon the result of which he basis his antagonism certainly do not show this. What they do is, to make clear a degree of insufficiency, which is far from what is necessary to sustain his criticism.

In order to make his argument tenable, he must proceed in one of two ways. He must demonstrate the views of those who maintain that a great many canals cannot be treated at all, and that a large number can only be treated partially, as being erroneous, or else that he can proceed in a manner, in the treatment of canals, that insures the desired tissue to be effected at every point, and not a point farther; and, moreover, that this can be done every time. Had the doctor selected a tooth in the jaw for canal treatment, and made an application of carbolic acid or zinc chloride, and could determine the requisite quantity to be applied that would insure this result, and then extracted the tooth so treated, and demonstrated that to have been done, then the experiment would have force.

But instead he proceeded quite differently. He took teeth out of the mouth, drilled an opening into the pulp-chamber, and in this fitted a glass tube. The end of the root is sealed. Into the tube a quantity of zinc chloride is poured, and the root surrounded with egg albumen. After a time the observation is made that the zinc chloride effects the albumen on the outside, and from this the attempt is made to prove one side of the controversy and disprove the other.

It is evident that in these experiments the conditions have not been observed to offer any positive or reliable basis for any antagonism or criticism to those views of which, Dr. Kirk says, Dr. Harlan is the originator or chief exponent.

It is curious to observe in his experiments that in proportion as any point appears valuable, so is there no confirmation of it. He states the objection to coagulants in canals to be a twofold one (meaning irritating coagulants). In the first place, such application seals the ends of the tubes, thus preventing the further entrance of any coagulant or antiseptic, and also of enclosing within a certain quantity of infected organic matter, which may, by its decomposition act subsequently as an irritant to the pericemental membrane. Later on he admits that he is unable to say whether coagulation invariably means sterilization.

Now, suppose we admit that the use of carbolic acid in canal work brings about entire coagulation, which fact, however, Dr. Kirk or no one else has ever shown, unless the point is that coagulation means sterilization, we fail to see any advantage in its use. But, on the other hand, such application may be disadvantageous should any decomposition arise.

Dr. Kirk answers himself when he quotes the following significant remarks of Dr. Atkinson: "He must go a little deeper and nominate the changes that occur between the food product and the chemical product,—that is, the result of the changes made by bringing the various substances together. There are affinities resident in the primal elements that have to be satisfied to bring about the changes we have recognized. We have not recognized the true theory so as to get at the alphabet of the molecular changes when we are considering the results as we deal with diseased tissue." In the application to the canal of such coagulant as espoused by Dr. Kirk, the first effect would be to coagulate a certain portion of the dentine, so much and no more, and after that it would not be a question of osmotic action.

Supposing, now, repeated applications are made, and they fulfil the condition of osmotic action. In what manner could we determine just how much of the surrounding structure became coagulated, or, in other words, even though applications to the canal act upon the principle of osmotic action, what, then, should prevent the cemented structure from being effected, and, in being effected, act as an irritant that might eventually prove very serious? Osmosis, then, which he takes particular care to emphasize, has nothing to do with the matter at hand, nor can we say that osmotic action takes place in the canal when in position in the mouth, because the conditions are entirely dissimilar to his experiments; and, finally, even should we establish osmosis, it is fraught with serious consequences.

Dr. Kirk once more quotes Dr. Atkinson. In a discussion concerning Dr. Harlan's paper, in which it appears the statement was made "that anything that will coagulate albumen is not fit to be used in the treatment of the root canal;" to which Dr. Atkinson replied that "chloride of zinc has repeatedly been introduced to preserve exposed pulps that has converted the entire pulp throughout the length of the canal into a hyaline coagulum that has proved just as good a filling as can be, and has stood from year to year, and without coloring the teeth or giving any indication of retrogressive movement whatever." If Dr. Kirk offers this in confirmation of his conclusions, it is very strange indeed. Strange, because he is very particular in admitting evidence, and strange that any one should apply chloride of zinc to an exposed pulp for its preservation.

Dr. Kirk advances upon a recruit to the army of "non-coagulants" which he is attacking, in the person of Dr. Ritter, of Utica, N. Y., although Dr. Ritter appears to be but half a recruit. Dr. Kirk finds fault with Dr. Ritter's procedure because that gentleman uses zinc chloride only in cases where the canals have been cleansed previously by a saponifying agent and *where the canal is large enough to permit of its successful working.* Now, he may permit us to say that is just the point precisely, "*where the canal is large enough to permit of its successful working,*" and he must establish the fact that irritating coagulants produce the result in dental work implied by his experiments in osmotic action, and not permit experiments in osmotic action to establish anything in the way of the action of irritating coagulants in pulpless teeth in the mouth.

The statement that the use of such coagulants as carbolic acid and zinc chloride has not only overwhelming clinical evidence in its favor, but that it is actually proper on theoretical grounds and fully in accord with the laws of osmosis, can be doubted with greater reason than it can be accepted. Particularly does this appear so from his experiments, and not only is the evidence overwhelming against the use of such coagulants, but the theoretical grounds, as well as the laws of osmosis, as far, at any rate, as his experiments relate, do not advance the question. Their coagulating property in a great number of pulp-canals can be urged scientifically against their use, and if the laws of osmosis can elucidate the problem, the experiments do not indicate it.

Dr. Kirk steps aside somewhat from the issues of his paper when he quotes some one concerning our educational methods. If he extends that as a hard thrust to any of his fellow practitioners, he may have overlooked the fact that it strikes just as hard home; and if he feels that our system of education is principally a process of mental stuffing, and that it is of far higher value to criticise evidence than accept all that is taught, and that much of what is taught is as yet an open question and accepted too blindly upon authority, it is equally as faulty to institute experiments that do not "demonstrate," and to accept such proceedings in any manner that carries the remotest sense of conviction with it.

